# A survey of fungal microbiota in airways of healthy volunteer subjects from Puglia (Apulia), Italy

**DOI:** 10.1186/s12879-019-3718-8

**Published:** 2019-01-22

**Authors:** Giovanna Elisiana Carpagnano, Antonia Susca, Giulia Scioscia, Donato Lacedonia, Grazia Cotugno, Piera Soccio, Sonia Santamaria, Onofrio Resta, Giuseppe Logrieco, Maria Pia Foschino Barbaro

**Affiliations:** 1 Department of Medical and Surgical Sciences, Institute of Respiratory Diseases, University of Foggia, Foggia, Italy; 2 Institute of Sciences of Food Production, National Research Council, Bari, Italy; 3 Department of Respiratory Diseases, University of Bari, Bari, Italy; 4 Medical student of the University of Bari, Bari, Italy

**Keywords:** Fungal microbiota, EBC, Respiratory contamination

## Abstract

**Background:**

The human respiratory tract represents the major portal of entry for numerous microorganisms, primarily those occurring as airborne particles such as viral and bacterial entities, or fungal spores. Microorganism characteristics coupled with the local host immune response will determine whether they will be cleared or adhere and colonize the airways leading to acute or chronic pulmonary disease. Like bacteria, fungi can cause severe lung diseases, but their infection rates are much lower. The lung microbiota is commonly sampled using relatively invasive bronchoscopic procedures. Exhaled breath condensate (EBC) collection offers a potentially less invasive alternative for lung microbiota sampling. This study tries to determine the composition of fungal communities in a cohort of healthy adult volunteer subjects from Puglia (Apulia), Italy.

**Methods:**

Fungi diversity in 27 EBC samples collected from Italian adult volunteers was investigated using conventional microbiological culturing and DNA sequencing approach.

**Results:**

Ten tested subjects (37,03%) turned out to present fungi in the EBC. We observed complex fungal communities, in which more than 10% of the isolated species are represented by *Aspergillus sydowii* (14,8%) and *Cladosporium spp* (11,11%). Three subjects that showed fungal presence in EBC have been diagnosed with a respiratory disease.

**Conclusions:**

We present a survey of an important scientific field in its early stages that is fungal contamination of airways of healthy subjects in a small geographic area. Furthermore, we interpreted our results to highlight the potential role of fungi in the context of respiratory diseases.

## Background

Within the last decade, many studies have highlighted the radical changes in the components of indoor and outdoor dust. Humans consistently face dermal, respiratory, and dietary exposures to these particles either indoors or outdoors.

A growing body of evidence from human and animal studies has revealed a link particularly between fungal exposure and lung diseases. This is not surprising, first, in consideration that we are exposed to the external air, that might include fungal spores through our airways [1] and secondly because fungi are ubiquitous in indoor and outdoor environments [2].

While many of these fungal spores are innocuous, some have the potential to germinate and cause invasive lung diseases [1]. The most known respiratory disease linked to fungi is asthma, but fungal spores can contribute to several other pathological conditions such as bronchial pulmonary allergic aspergillosis (ABPA), pneumonia and lung cancer [1, 3]. *Aspergillus* genus includes some of the most dangerous toxigen fungi common in the Mediterranean environment, able to colonize different crops, including maize, grapes and dried fruits [4–7], and to produce mycotoxins, such as aflatoxin, ochratoxin A and fumonisins [8], when host plants are stressed by extreme temperature or moisture conditions, poor soil fertility or insect damage. Our group recently described the presence of *Aspergillus* species in lung cancer patients using as a matrix coming from the airways, the exhaled breath condensate sample already validated in the study of airways microbiota [3]. Other fungi were also found in our oncologic population as *Aspergillus ochraceus* or *Penicillium* spp., whose possible role in the development of cancer and other airways diseases is not yet known. Notwithstanding the recognized dangers for human health of *Aspergillus* and other fungi, our knowledge showed an absence of contamination of airways [3] and no studies are available that prove the possible contamination of airways of healthy subjects with outdoor fungal spores. Furthermore, there is still limited evidence of possible physiological impact of fungal contamination on the respiratory system [9].

Moreover, epidemiological studies often rely on broad microbiota exposure but fail to identify the taxonomic composition of the microbial community [10]. With this study, we want to give a preliminary contribution to this lacking field of research, giving a view of the incidence and nature of possible fungal contaminations in healthy subjects from Puglia. Toward this goal, we tried to achieve a better understanding of species/taxon diversity and population dynamics of the fungal microbial community present outdoors in the Puglia region, giving a survey of fungal microbiota of airways of healthy volunteer subjects.

## Methods

### Characteristics of enrolled volunteers

Twenty-seven consecutive white Caucasic Italian volunteers (mean age: 46.3 ± 15.3 years; 12 male; BMI: 27.2 kg/m^2^; 10 smokers; 25 living in industrialized areas and 2 in rural areas or involved all day in rural activity) were enrolled in September 2016 during the annual public regional meetings “Fiera del Levante”, held in the city of Bari.

The study was approved by the institutional ethics committee of the University of Foggia (institutional review board approval number 17/CE/2014). All subjects were informed of the purpose of the study; after signing the informed consent form, anthropometric, physiological, clinical data and exhaled breath condensate were collected.

### EBC collection

The exhaled breath condensate was collected in one sitting from each subject, by using a condenser, which allowed for the non-invasive collection of non-gaseous components of the expiratory air (Ecoscreen Jaeger, Wurzburg, Germany). One (1) mL of EBC was collected from each volunteer and spread onto Petri dishes containing selective medium for yeasts and moulds, Dichloran Rose-Bengal Chloramphenicol Agar (DRBC, Oxoid), and then incubated at 25 °C for 7 days in darkness.

### Fungal cultures and species identification

Fungal genera and species were determined observing fungal morphological features, according to the taxonomic keys of Klich [11]. Mycelium of representative colonies was transferred on dishes containing Potato Dextrose Agar (PDA, Oxoid) and incubated at 25 °C for an additional 5 days. The mycelia from single colonies were scraped and collected in 1,5 mL tubes for DNA extraction.

### DNA extraction, PCR amplification and sequencing

DNA extraction was performed using the Wizard® Magnetic DNA Purification System for Food kit, that uses paramagnetic particles, according to the manufacturer’s protocol. DNA was recovered and dissolved in sterile water. PCR amplifications of β-tubulin or ITS gene were set up using approximately 20 ng of fungal DNA template. Reactions were performed using primer pair Bt2a/Bt2b [12] and ITS4/ITS4 [13] according to the following conditions: 5 min at 94 °C; 50s at 94 °C, 50 s at 59 °C, 1 min at 72 °C for 35 cycles; 7 min at 72 °C followed by cooling at 4 °C. PCR amplicons were assessed by agarose gel electrophoresis and purified with enzymatic mixture EXO/FastAP (Exonuclease I, FastAP thermosensitive alkaline phosphatase, Thermo Scientific, Lithuania, Europe). Sequence reactions were performed using a BigDye Terminator v3.1 Cycle Sequencing Ready Reaction Kit for both strands, purified by gel filtration through Sephadex G-50 (5%) (Amersham Pharmacia Biotech, Piscataway, NJ, USA), and then analyzed on the 3730xl DNA Analyzer (Applied Biosystems, Foster City, CA, USA). DNA sequences were determined by Sequencing Analysis 5.2 software (Applied Biosystems). Sequence similarity searches for species identification were performed for each strain against the non-redundant database maintained by the National Center for Biotechnology Information using the BLAST algorithm (http://www.ncbi.nlm.nih.gov).

### Statistical analysis

In order to assess the association between categorical variables such as sex, smoking habit or fungi positivity in the EBC the chi square test (or Fisher’s exact test, when necessary) were calculated. The Student’s T-test was used for independent samples in order to assess the differences in continuous variables (sex, age, BMI, smoking habit, pack years, area of residence, job) between positive and negative airways contamination. A *p* value of < 0.05 was considered statistically significant.

## Results

The demographic and clinical data of study subjects are summarized in Table 1.

**Table 1:** Anthropometric, clinical and microbiological data from volunteer subjects

		Subjects
Number		27
Age (years)		46.3 ± 15.3
Sex	Male	12
BMI		27 kg/m^2^
Smoke habit:	Smokers/Ex-Smokers	15
	Non smokers	12
Area of living	Industrialized areas	25
	Rural areas	2
Job	Involved all day in rural activity	2
	Not involved all day in rural activity	25
Respiratory diseases:	COPD	2
	Asthma	1

The study detected the presence of the following fungal species in the EBC samples: *Aspergillus sydowii* (14,8%), *Cladosporium spp (11,11%), Cladosporium herbarum* (3,7%), *Penicillium brevicompactum* (3,7%), *Penicililum expansum* (3,7%), *Penicillium glabrum* (3,7%), *Penicillium olsonii* (3,7%), *Penicillium bilaiae* (3,7%), *Alternaria infectoria* (3,7%), *Alternaria alternate* (7,4) (Table 2).

**Table 2:** Fungal species isolated from EBC for each healthy volunteer enrolled

Subjects	Fungi isolated
1	*Cladosporium* spp
2	*Aspergillus sydowii*
3	*Aspergillus sydowii* *Penicillium brevicompactum* *Cladosporium sp*
4	*Penicillium expansum* *Cladosporium spp*
5	*Alternaria infectoria* *Alternaria alternata*
6	*Aspergillus sydowii*
7	*Penicillium glabrum*
8	*Alternaria alternata*
9	*Penicillium bilaiae* *Aspergillus sydowii*
10	*Penicillium olsonii* *Cladosporium herbarum*

Among tested subjects, 10 (37,03%) turned out to be fungi contaminated in the EBC: 5 (18,51%) were contaminated by more than one fungi (Table 2). Three (3) of 10 (30%) subjects, that showed fungal contamination, were affected by lung diseases [2 (20%) by COPD and 1 (10%) by asthma], while others were anamnestically healthy.

When analyzing subjects with fungal positivity in their EBC, no difference was found according to sex, age, BMI, smoking habit, pack years, area of residence, job (*p* > 0.05).

## Discussion

Previous studies with sequencing different fungal genera in indoor dust and outdoor air samples showed that significant proportions of *Aureobasidium* and *Leptosphaerulina* along with some contribution from *Cryptococcus*, *Epicoccum*, *Aspergillus* and the human commensal *Malassezia* [10], and that the indoor air microbial communities are thought to be a function of dispersal from the outdoors, and growth and resuspension from the indoor environment [14]. Visagie et al. [15] and Flannigan et al. [16] listed 100 fungal species common in indoor environments, including *A. fumigatus, A. sydowii, P. brevicompactum* and *P. citrinum*, classified as common in collected house dust, but the origin of common indoor species is difficult to determine.

The present study is the first to take a real picture of the incidence and nature of fungal contamination in healthy subjects from the Puglia Region of Italy. We tested fungal microbiota of exhaled breath condensate of healthy subjects and used the DNA sequencing approach for fungal species identification. ITS is the most commonly sequenced gene for fungi and was recently accepted as the official DNA barcode [17], but it does not distinguish among all species, because some species share identical sequences [18–21], even though it provides valuable information on sectional classification and often provides enough information for species identification. In order to compensate for the lack of variability in ITS, we also used BenA, as a secondary identification marker.

The presence of moulds was detected in 37,03% of enrolled healthy subjects (*Aspergillus sydowii, Cladosporium spp, Cladosporium herbarum, Penicillum brevicompactum, Penicillum expansum, Penicillum glabrum, Penicillum olsonii, Penicillum bilaiae, Alternaria infectoria, Alternaria alternata).* The fungal positivity in airways didn’t correlate with any analysed variables (sex, age, BMI, smoking habit, pack years, area of residence, job).

However, the fungal contamination of airways of healthy subjects that we reported in this study was very high (37,03%) and, also supposing a high concentration of fungi in ambient air, we were surprised that almost half of the subjects enrolled had contaminated airways. This data was unexpected, especially in consideration that in our previous study [3] we didn’t find fungal contamination in healthy subjects. Surely, the enrollment conditions were completely different because in the previous study subjects were taken from the outpatient clinic and samples were collected in the clinic while, in this real life study, subjects were enrolled during the annual public regional meetings “Fiera del Levante”, held in the city of Bari that subjects visited as tourists.

*Aspergillus sydowii* is one of the most common species in collected samples and the species is generally considered as widespread. The species is often isolated from soil [22], and is very common on mouldy gypsum wallboard, dust, paint and various foods [16, 23, 24] and is commonly found in marine environments where it acts as an opportunistic pathogen of sea corals [25–29]. The source or origin of this species is still unknown, even though most studies indicate it as a terrestrial soil-borne fungus and shows its ability to grow in such a wide range of niches, suggesting the need for further studies that might help in understanding their possible role in underestimated pathologies.

However, the possibility of a high outdoor contamination of fungi is coherent with the season. Enrollment of patients took place in September, one of the hottest and most humid months in southern Italy and we know that fungal spore counts properly peaked during warm months [30].

Furthermore, the days of enrollment were particularly windy, a climatic condition that further contributes to the large diffusion of fungal spores.

However, it is virtually impossible in this study to determine the fact that the inhalation of fungal spores surely leads to fungal presence in airways after exposition. In the same manner the presence of fungi does not automatically determine future contamination. Indeed, this study has been designed just to take a survey of fungal microbiome in airways of healthy subjects from the Puglia Region and therefore enrolled patients just once.

A limit of the study was not to have repeated the collection of exhaled breath condensate and subsequent fungal analysis of this airway’s sample from healthy volunteers a short time after exposure. It would be very useful to see what happens after 1 day, 1 week-end, 1 month from exposure to a contaminated outdoor environment.

Another important limit of this study, due to the conditions previously explained, was to have collected clinical data of patients only with an anamnesis. Subjects were volunteers who came to our respiratory stand at the “Fiera del Levante” just to test their airways contamination.

A spirometer or other clinical instruments were not available in order to test lung function and to diagnose possible respiratory diseases. Therefore we were only able to identify a respiratory condition that might justify fungal contamination in airways.

An important point of this study was to have used a non-invasive method to analyse the airways of healthy subjects that otherwise would not have undergone more invasive techniques of sampling airways. Our group previously demonstrated the suitability of the EBC as non-invasive sample for the study of fungal microbiome of airways and this study further confirms its value [31].

We were unable to find any correlation between fungi positivity and sex, age, BMI, smoking habit, pack years, area of residence, or job. However, the number of subjects enrolled in this study was low and justified our results, which we intend to verify on a larger population.

Furthermore, important analyses should also be addressed to identify the fungal genotypes isolated, assess their ability to produce toxins, and above all, to evaluate the effective presence in human fluids, such as the EBC, of mycotoxins, potentially produced by isolated *Aspergillus* species or other fungi. This was just a preliminary study that will be followed by a genomic and epigenomic characterization and mycotoxin analysis of airways of healthy subjects.

Methods for taxonomic identification of microbial communities through metagenomics approaches to DNA sequencing are rapidly gaining importance in fungal biodiversity research, allowing both generation of barcode markers and identification of isolates to species level [32], but reference databases are mostly incomplete, and, mostly developed for purposes other than the study of the relation of the environmental microbiome to human physiologic or health outcomes. Thus, EBC has the potential to study a more complete view of fungal microbial communities, or even previously unstudied individual taxa that may influence human health. However, the EBC microbiota may still be an interesting avenue of study despite the fact that the small quantities of bacterial DNA in these samples leave them more vulnerable to contamination, and any future studies would have to be designed with this in mind.

## Conclusions

We isolated fungi from airways of 37,03% of healthy volunteer subjects visiting a public regional meeting “Fiera del Levante”. Data collected contributed to outlining a real survey of fungal microbiota in airways of healthy subjects from Puglia, but the study needs to be confirmed on a larger population for both the potential and limitations for this type of exposure assessment. A deeper investigation should be set up to understand the source of fungal spores in airways, lower or upper airways, and therefore their potential effects on human health.

Furthermore, there is the possibility that the presence of some spores reflect the normal air flora present in the ambient air at the time of sampling and that therefore it would be interesting to sample the ambient air to support this hypothesis. However, this was an unexpected finding that now opens the way for new interesting research studies that absolutely will include a careful analysis of the air we breathe. However, the presence of fungi in airways leads us to support the necessity of modifying our previous knowledge on the normal flora. Furthermore, it is important to start thinking about whether these fungi find favorable conditions in airways for germination and subsequent production of mycotoxin, often dangerous for human health. The complete airways flora of healthy subjects should also be considered and could become very important for prevention programs.

## Abbreviations

ABPA: bronchial pulmonary allergic aspergillosis
BMI: Body Mass Index
COPD: chronic obstructive pulmonary disease
DRBC: Dichloran Rose-Bengal Chloramphenicol Agar
EBC: exhaled breath condensate
PDA: Potato Dextrose Agar
